# Spectral effect of streetlamps on urban trees: A simulated study on tissue water, nitrogen, and carbohydrate contents in maple and oak

**DOI:** 10.1371/journal.pone.0248463

**Published:** 2021-03-25

**Authors:** Ping Liu, Baohui Cao, Yutao Wang, Zhongping Wei, Jingfeng Ye, Hongxu Wei

**Affiliations:** 1 College of Forestry, Shenyang Agricultural University, Shenyang, Liaoning, China; 2 Liaoning Academy of Forestry Science, Shenyang, Liaoning, China; 3 Northeast Institute of Geography and Agroecology, Chinese Academy of Sciences, Changchun, Jilin, China; 4 University of Chinese Academy of Sciences, Beijing, China; Qingdao Agricultural University, CHINA

## Abstract

Streetlamps enforce night lighting on urban forest trees, but scarce information is available concerning the ecophysiological performance of street trees under these conditions. In this study, maple (*Acer truncatum* Bunge) and oak (*Quercus mongolica* Fisch. ex Ledeb.) seedlings were cultured with simulated exposure to streetlamp spectra in white (red/green/blue, 7.7:1.0:2.2) and red plus blue (RB; red/green/blue, 4.6:0.0:1.0) lights with photosynthetic photon flux rate of 80 μmol m^-2^ s^-1^ in a 18-h photoperiod. Nitrogen (N) was loaded through 15 weekly applications to an amount of 80 mg N plant^-1^ to mimic the mineral N deposition to landscape trees. Variables of biomass, carbohydrate accumulation, N and water contents were rarely found difference between the two LED-spectra treatments for both species. Compared to the un-lighted control, the RB spectrum lowered N concentration in oak seedlings and water content in maple seedlings. The white light spectrum resulted in an increase of starch concentration. Carbohydrate concentration had a positive relationship with biomass and N content across two species but a negative relationship with water content in maple seedlings. Overall, streetlamp-lights imposed effects on tree growth by a prolonged photoperiod instead of specific spectrum. Maple had a strong response of water uptake to streetlamp lighting at the cost of carbohydrate consumption, but oak had scarce demand of water-use for growth.

## Introduction

Urbanization is a progress of highly attracting and utilizing water resources by the continuously concentrating population in built-up areas. The tradeoff between population growth and water shortage in cities is hard to be mediated when the complexity for precipitation frequency increases with climate change [1, 2]. To purchase a higher water use efficiency is one of the core missions for to meet low impact development in urban forest planning [3, 4].

Urban ecosystems are closely accompanied by streetlamps whose light prolongs illumination period for trees with various lighting spectra. Urban forest trees are frontiers subjected to streetlamp lighting. Tree phenology obtained extension due to continuous lighting at night [5]. Spectrum of lighting at night is a critical factor that shapes tree growth and physiology [6–9]. Lighting spectrum can also impose impact on plant moisture content although few observations have been documented on trees [10–12]. The increase of water use efficiency can be accompanied by the decline of carbohydrate accumulation, especially for starch, due to lowered stomatal conductance [13, 14]. In contrast, artificial illumination at night promotes non-structural carbohydrates (NSCs) metabolization [8, 9]. This is the reason why night-light-exposed tree growth is enhanced [15–17]. Continuous light spectrum can further modify biomass production and allocation through regulating carbohydrate metabolism [6–9]. To figure out the relationship between carbohydrate metabolism and other physiological processes is the key to account for the effect of streetlamp spectrum on water content in urban forest trees.

The spectra of lights changed with components of red (600–700 nm), green (500–600 nm), and blue (400–500 nm) lights. The content of photosynthetic pigments and chloroplasts in grana can be enhanced by the spectrum with red and blue lights [18]. The high proportion of red light in the wavelength with low blue light was found to bring about restriction on photosynthesis, carboxylation, electron transport, triose phosphate use, and leaf thickness [19, 20]. These pre-conditions, however, cannot induce any changes in carbohydrate concentration by the monochromic spectrum in the wavelength of red or blue lights. Instead, a multi-lights spectrum with high-red proportion can induce low glucose content but result in high starch accumulation [21–23]. The ratio of red to green lights in understory sunlight determined the consumption of foliar starch [24]. The spectrum with high red/green light-ratio promoted the hydrolyzation of starch in roots [6] and sugar in stem [8]. Most spectra tested in current studies were designed with the purpose to promote the potential efficiency of plant culture. Few findings were conducted for the analysis of streetlamp-spectral effect on NSCs metabolism in urban trees.

Nitrogen (N) is an essential element that is responsible for many physiological processes in forest crops exposed to different spectra [6, 7, 9, 25]. In a city area, both NH_4_^+^ and NO_3_^-^ are imposing loads through bulk wet and dry depositions to urban forest ecosystem [26]. The impact of N input is an inevitable factor that modify growing condition for urban trees. Any spectra that promoted plant growth and biomass accumulation would result in N dilution or even depletion due to a decrease in N concentration [6–8]. Given that many nitrogenous compounds are synthesized at the expense of starch consumption by hydrolyzing to soluble sugars [27], data across species together indicated a positive relationship between sugar and N concentrations [8, 27, 28]. Nitrogenous enzyme, e.g. sucrose synthase, will further account for the conversion of sucrose to polysaccharides [28]. These conversion-chains from starch through sugar to polysaccharides can be interfered by the change of lighting spectra as unchanged starch accumulation and over-consumption of sugars [8]. However, more evidence is required to detect the changes of starch and sugar concentrations and their relationships with N concentration in more tree species.

**Fig 1:**
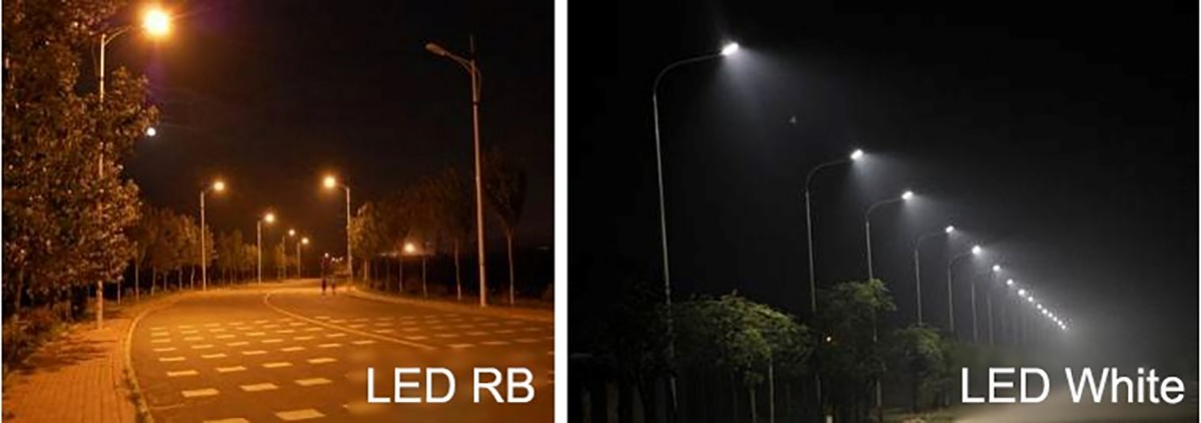
Appearance of lighting of red plus blue (RB) and white lights from streetlamps using light-emitting diode (LED) as source of lighting in Shenyang City, Liaoning Province, Northeast China.

Lighting from streetlamps is mainly provided by high-pressure sodium (HPS) lamps [5] and white-color bulbs [29] emitting combined red- and blue-light wavelengths and white light wavelength, respectively. These two types of spectra can both be found in streetlamp lights at Shenyang city, Liaoning Province, Northeast China (Fig 1). Therefore, the response of urban forest tree species to streetlamps in Shenyang City can support deeper detection on physiological mechanism in a natural laboratory. The objective of the current study was to determine the responses of NSCs, N status, and water uptake across urban forest tree species in Shenyang to different spectra from streetlamps and their relationships. We hypothesized that starch concentration had a (1) negative relationship with N concentration (2) but a positive relationship with water uptake, (3) while relationships with sugars were adverse, respectively.

## Materials and methods

### Study site

This study was conducted in Shenyang City, Liaoning Province, Northeast China. Shenyang locates in the transitional belt between Changbai Mountains and the alluvial plain of the Liaohe River (41°11’–42°17’ N, 122°21’–123°48’ E). Shenyang is the capital city of Liaoning province with 8.3 million of permanent residents distributed in an area of 6.3 million km^2^ built-up region up to 2018. Shenyang locates in a zone of semihumid temperate continental climate with annual average temperature of 6.2–9.7°C in a range of -32.9°C and 38.4°C. Annual rainfall in Shenyang ranged between 600 and 800 mm with historical maximum precipitation of 716.2 mm in built-up zone. Yearly frost-free periods lasted for 155–180 d. All these climatic data were cited from the document from 1951 to 2018 [30].

### Urban forest species

There were generally five main types of urban forests in Shenyang [31]. They were ecological and public welfare forest (EF), attached forest (AF), landscape and relaxation forest (LF), production and management forest (PF), and road forest (RF). Area and stem density for these five types of forests were investigated to be: EF, 47.1 km^2^ and 653 ± 117 individuals ha^-1^; AF, 22.7 km^2^ and 375 ± 55 individuals ha^-1^; LF, 12.3 km^2^ and 502 ± 42 individuals ha^-1^; PF, 10.9 km^2^ and 905 ± 133 individuals ha^-1^; RF, 8.4 km^2^ and 279 ± 47 individuals ha^-1^ [31]. Maple (*Acer truncatum* Bunge) and oak (*Quercus mongolica* Fisch. ex Ledeb.) are two of the main landscape species used in AF, which were frequently found in urban forest parks and arboretums. In Shenyang, broadleaf species of *Populus canadensis* and *Ulmus pumila* were also used in landscape greening for AF [31], but their growing rates are too high under continuous lighting to be used in a simulated experiment. In contrast, growing rate of *Pinus tabulaeformis*, which is also a popular landscape species used in AF at Shenyang [31], was extremely low with slothful change of NSCs [32]. Both oak and maple species have a moderate growing rate that is suitable for study in a controlled environment. They both have a flexible pattern for allocation, storage, and remobilization of NSCs to mediate physiological response to the environment [33, 34]. Therefore, maple and oak were taken as the species that were tested in this study.

### Streetlamp lighting quality assessment

**Fig 2:**
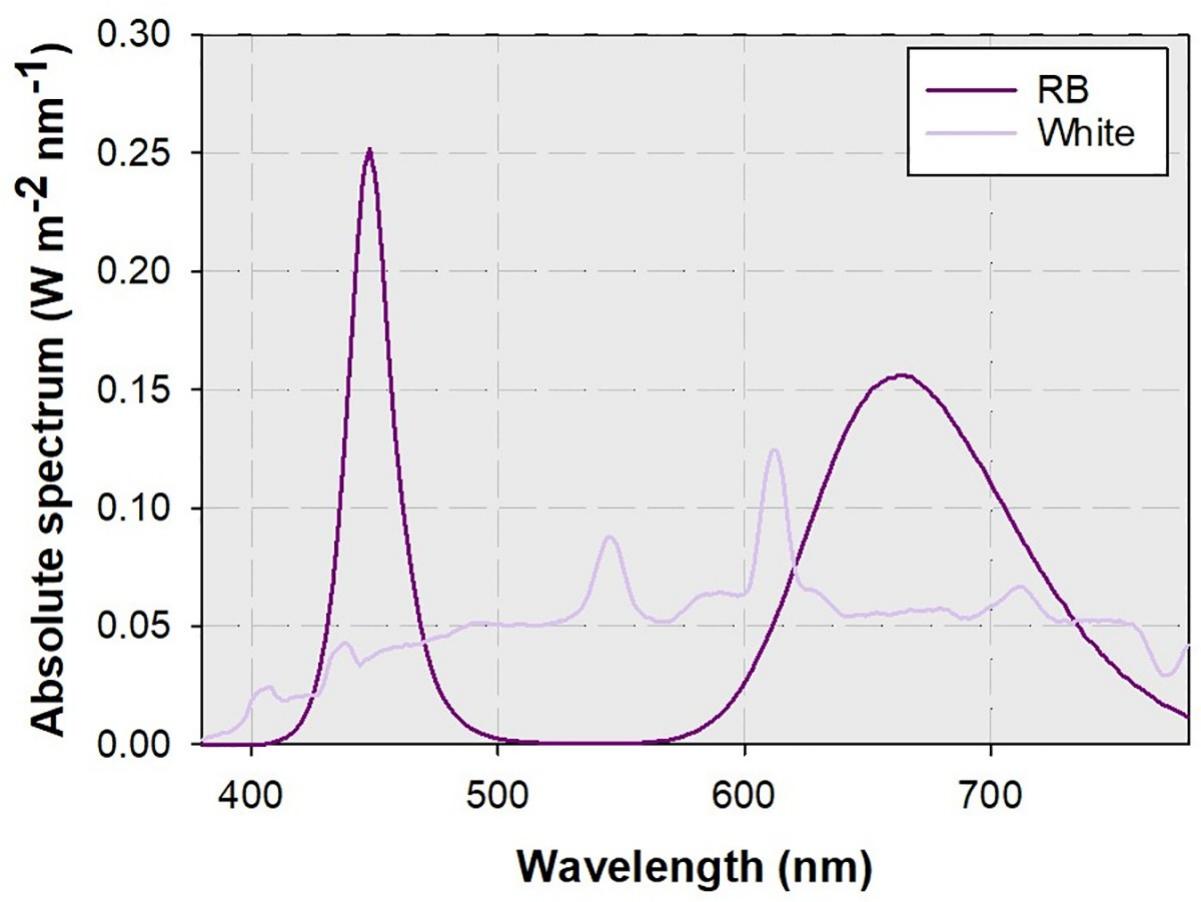
Spectral values of continuous wavelength for simulated streetlamp spectra of red plus blue (RB) and white lights.

Streetlamps using LED as source emitting RB and white lights were chosen as the source of variance for the effect of lighting spectra (Fig 2). Streetlamp lighting characteristics were measured during the summertime in July 2017. The permission for location was issued by the authority of College of Forestry of Shenyang Agricultural University. At nighttime during 19:00 and 24:00 pm following a sunny daytime with no rain or blowing, streetlamps that were built in the landscape of maple and oak populations were chosen as the objects to measure lighting quality. The photosynthetic photon flux rate (PPFD) and ratio of wavelengths in red (600–700 nm), green (500–600 nm), and blue (400–500 nm) lights for streetlamps was measured using a portable meter (EverFine PLA-20, Yuanfang Elect. S&T Inc., Hangzhou, China). The ratio of red to far-red lights was measured to be 0.8. A ladder and an elongated pole were used to hold the device to touch the lighting range as close as to 50 cm for metering the real-time PPFD. The place to maintain the device needs to be close to the tip of maple and oak tree crowns that were exposed to the streetlamps with no barriers. Ten cycles of monitoring were recorded to calculate the mean value for one time and at least five positions were used to be measured for averaged lighting quality of maple or oak tree crowns.

### Nitrogen input assessment

According to the manager for the urban forest park where we investigated streetlamp lighting for maple and oak populations, no fertilizers were inputted to trees for at least 5 years up to 2017. Therefore, we took mineral N deposition as the main source of N input to the trees. A recent investigation revealed that seasonal N input to local forests were estimated at rates of 4.0 and 4.3 kg ha^-1^ a^-1^ as nitrate and ammonium forms during summertime, respectively [26]. Therefore, a total rate of 8.3 kg ha^-1^ a^-1^ was assumed to inputted to maple and oak trees. Tree density in the AF type was counted to be 375 stems ha^-1^ [31]. Therefore, a mature tree received a dose of 22.13 g N ha^-1^ a^-1^ through deposition. The C density for the AF type trees was estimated to be 50.17 ton ha^-1^ [31] which led to an amount of 0.13 ton tree^-1^ ha^-1^. As we used juvenile seedlings as materials in this study instead of the mature tree to quantify the whole-tree responses, the annual N input through deposition was lessened according to the scale of C in a seedling relative to that in a mature tree. The whole-plant biomass for maple and oak trees was measured to be 2.71 g per individual using samples of 10 one-year-old seedlings per species that were oven-dried at 70°C for 72 h. Vashum and Jayakumar [35] made a literature summary and concluded that C concentration of a tree can be 47.5% (45–50%) of the dry biomass. It can be further calculated that tree seedling equalizing to the C storage of 482.72 g ha^-1^, which was 99.64% lower than that in a mature tree per ha. Using this ratio, the theoretical N input to a seedling from deposition was calculated to be 79.86 mg N individual^-1^. We chose the amount of 80.0 mg N individual^-1^ as the rate to feed our seedlings. We did not test soils of the trees in the field measurement hence the amount of N input was hypothesized to be fully absorbed by maple and oak seedlings with a very low risk to induce N deficiency or excess.

### Experimental layout

**Fig 3:**
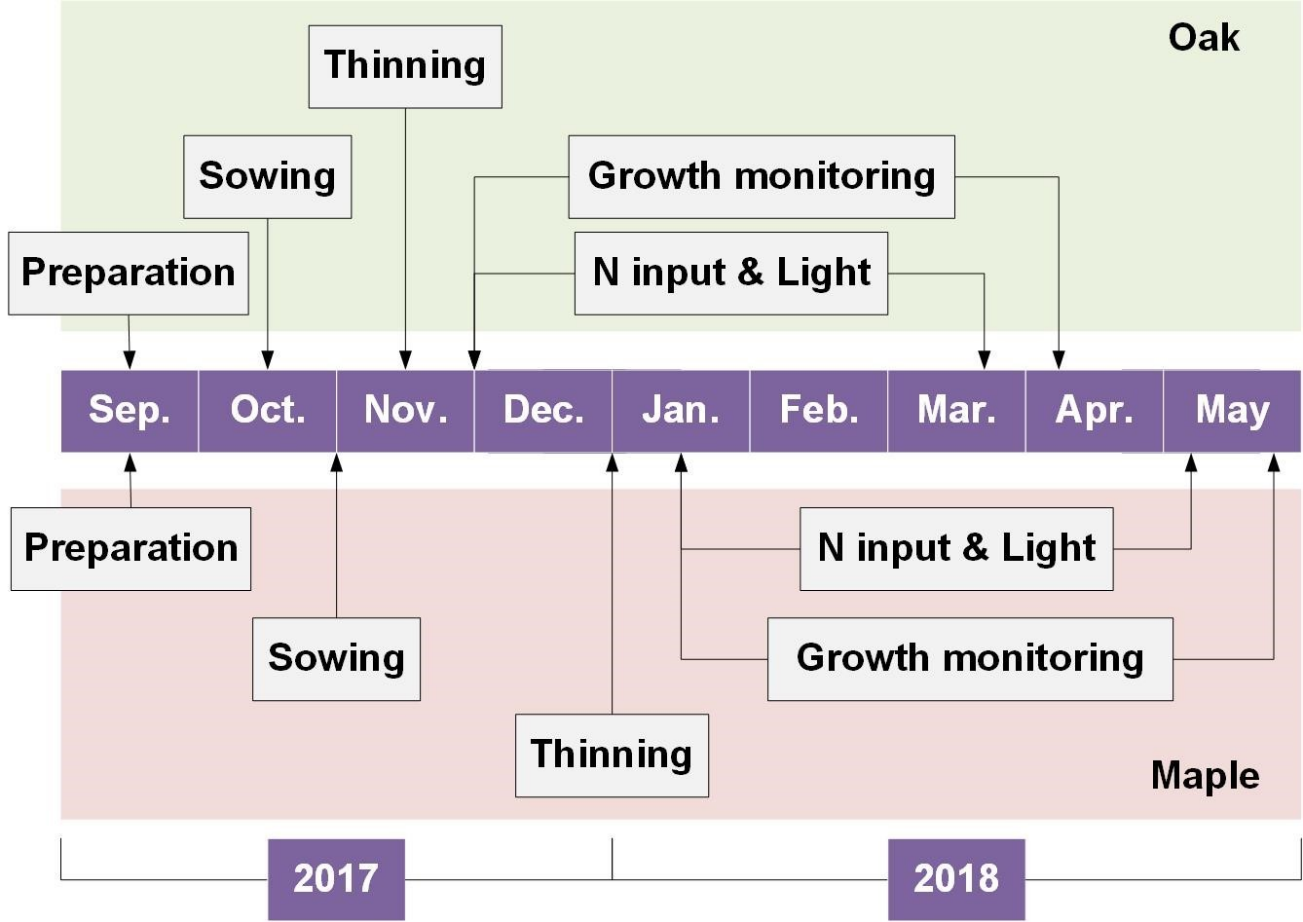
Arrangement of the whole experiment process.

Monthly details about experimental manipulations throughout the experiment are shown in Fig 3. Seeds were collected from mature trees in a remote rural region of Shenyang city in September 2017. Collected seeds were steamed using dimethyl dichlorovinyl phosphate for 24 h to eliminate living eggs of bugs and pests that may attack grown-up seedlings. Steamed seeds were soaked using warm water at the temperature of 30–40°C for 24h then removed out all dead ones floating over the water surface. The rest of seeds were placed on the surface of a moist towel for germination which was ended up for sowing as about 30% of seeds that had shown initial embryos. This percent of the exhibition of embryo is a practical sign for the transplant of germinated maple and oak seeds as a solid prediction of continuously developing and growing according to the local experience. All seeds were sown to planting trays with 32 cavities (13 cm deep, 7 cm top-diameter, and 212 cm^3^ volume) in a 4 × 8 arrangement. Cavities were filled with commercial substrates mixed by peat, spent-mushroom residue, and perlite in a volumetric proportion of 55:20:25 (Mashiro-Dust^TM^, Zhiluntuowei A&F S&T Ltd., Changchun, China). Three to four seeds were sown in one cavity which was then covered by substrates to a depth of 3 cm and watered to the full capacity. Half a month after sowing, seedlings started to germinate while the subsequent diameter growth for oak seedlings was faster than that for maple seedlings. Another half month later, seedlings were thinned to leave one individual per cavity when oak seedlings grew to the height of 7–10 cm and root-collar diameter (RCD) of 2–3 mm (Fig 3). At the same time, maple seedlings grew to have 7–10 cm height and ~1.5mm RCD. When thinning, about 70% of maple seedlings had grown to reach the phenological stage that can be coded by the Bilogische Bundesanstalt Bundessortenamt and Chemical Industry (BBCH) scale as a value of 11 with the rest and most oak seedlings grew to reach the BBCH coding by a value of 9 [36].

The layout of lighting treatment was conducted in the laboratory of forest silviculture of Shenyang Agricultural University. The streetlamp spectrum was stimulated by that emitted by LED panels (0.4 m × 1.2 m, width × length). Two artificial lighting spectra from simulated streetlamp lights were set as the treatments with an unlighted control. Controlled seedlings were subjected to the shaded sunlight at the average PPFD of 80 μmol m^-2^ s^-1^ in the natural photoperiod. Three LED panels per spectrum were fixed to the three chamber spaces in an ironic frame in the project area of 0.4 m × 1.2 m (width × length). In each chamber space, the PPFD, 50cm beneath the panel, was set to be 80 μmol m^-2^ s^-1^ by adjusting electric currency to mimic that for street tree canopy. The white light spectrum was measured to have a wavelength ratio of 7.7:1.0:2.2 for red, green, and blue lights, respectively; while the RB light spectrum was measured to have another wavelength ratio of 4.6:0.0:1.0. LED lighting was turned on at 6:00 am and switched off at 24:00 pm to supply an 18-h photoperiod. The lighting from 6:00 am to 18:00 pm was set to mimic the daytime lighting with sufficient PPFD and that from 19:00 pm to 24:00 pm was set to mimic the nighttime lighting given by streetlamps. During the whole period of LED lighting, the PPFD was kept constantly to be 80 μmol m^-2^ s^-1^ 50cm beneath the panel. Every LED panel was accompanied by two planting trays with each for a species as maple or oak seedlings which were together assigned as an experimental unit. Every unit was replicated for three times as six trays. Because initial growth of maple seedlings was slower than that of oak seedlings, the LED lighting was employed in December 2017 for oak and in January 2018 for maple (Fig 3). During the experiment, the temperature for trayed seedlings was monitored to fall in a range of 25–30°C.

Trayed seedlings were watered by a sub-irrigation system where seedlings were watered twice a week. Nitrogen was loaded to seedlings using nutritional solutions with N-abundance of 12% once a week to an amount of 80 mg N seedling^-1^ to mimic the deposited N input. All seedlings were measured for height and RCD weekly since the first week of lighting commencement up to the 17th week. After a time of 15 weeks of N delivery, oak and maple seedlings were terminated for lighting and received sampling in late March and early May in 2018, respectively. At the end of experiment, sampled seedlings were divided into shoot and root parts and measured for fresh weight and dry weight after oven-dried at 70°C for 72 h. Water content ratio was calculated as the water content difference (fresh weight minus dry weight) divided by dry weight. Dried samples were mixed together for shoot and root parts separately to be a whole-plant bulk, which was determined for N concentration using the Kjeldahl method [16, 17, 25] and for NSC using the colorimetric method with a spectrophotometer at the wavelength of 490 nm [37, 38]. Briefly, a 0.5-g dried sample was digested in hydrogen peroxide (H_2_O_2_) and sulfuric acid (H_2_SO_4_), diluted to 50 mL, and used for N determination. Another 0.5-g dried sample was dissolved in 80% ethanol (v/v), diluted to 50 mL of distilled water, heat by boiling at 100°C for 1 h, centrifuged at 5,000 rpm for 15 min at 4°C, and used for determination using a spectrophotometer at the wavelength of 490 nm.

### Statistical analysis

Nitrogen uptake efficiency (*NUE*) was calculated as whole-plant N content divided by delivered N amount (80 mg N plant^-1^). Nitrogen utilization index (*NUI*) for biomass and carbohydrate was calculated as whole-plant biomass and carbohydrate (soluble sugars, starch, and NSC) contents divided by N concentration.

Data were analyzed using SAS software (STS Institute, Cary, NC, USA). Normal distribution was tested for data of all variables and the log-transformation was used when it was necessary to meet the normality. A split-block design was used as a two-way ANOVA with species variation (maple vs oak) within the main block and three lighting regimes (control, RB, and white) within the sub-block in three combined replicates (species × light). The arrangement of lighting frames was set as the random factor as to eliminate the edge effect originated by the arrangement of main block. When significant effect was detected, results were arranged and compared as a one-way ANOVA involving all six combined treatments (2 species × 3 light spectra). Otherwise, results were compared according to Tukey test in response to the significant single effect from species (*n* = 9) or lighting spectra (*n* = 6). Vector analysis was employed to characterize nutritional status in LED spectra relative to the control for the two species. Nomographs were plotted according to the method by former studies [39–41]. Pearson correlation was employed to detect the relationship between any pair of whole-plant variables among biomass, carbohydrates (starch, soluble sugar, and NSC) concentrations (weight per dry mass) and contents (concentration × dry mass), N concentration and content, and water content. Principle component analysis (PCA) was employed to detect grouped relationships among variables. Eigenvalues for PCA were analyzed by the Princomp procedure in SAS software and plotting was finished by SigmaPlot v.14.0 software (Systat Software, Inc., San Jose, CA, USA).

## Results

### Height and diameter growth

**Fig 4:**
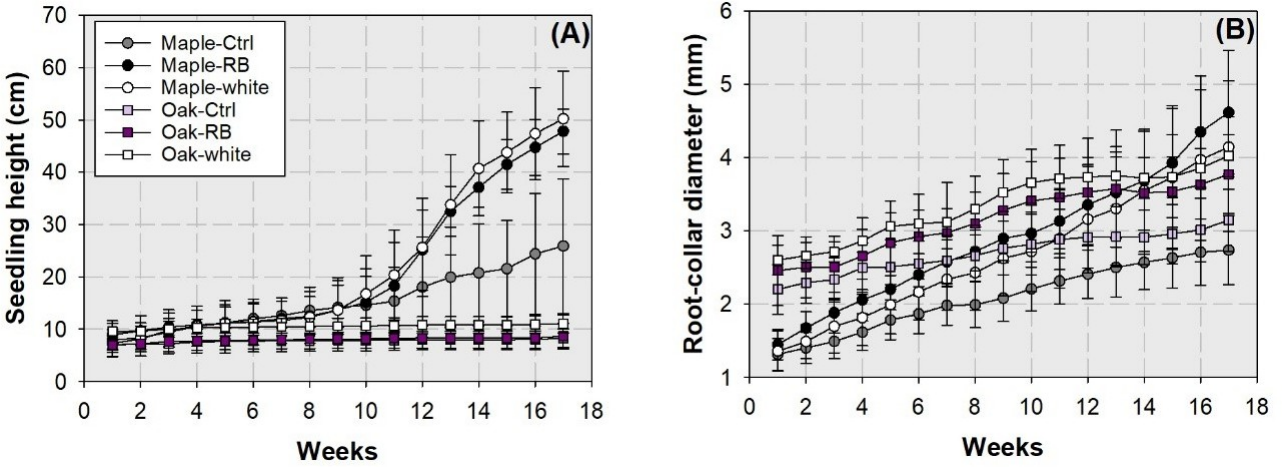
Dynamic growth in height (A) and root-collar diameter (B) in maple (*Acer truncatum* Bunge) and oak (*Quercus mongolica* Fisch. ex Ledeb.) seedlings exposed to simulated streetlamp spectra of red plus blue (RB) and white lights with an un-lighted control. Error bars present standard errors.

Factors of species variation and streetlamp spectra did not have any combined effects on seedling height growth until 13 weeks after transplant (Fig 4A). Since this week on, maple seedlings had higher height in the RB and white light spectra, followed by that in controlled maple seedlings. Seedling height in oak seedlings was generally lower than that in maple seedlings. No statistical difference was found for seedling height among oak seedlings subjected to any of the spectra. Oak seedlings in control and the RB spectrum had the lowest height in about 8 cm.

The factor of species variation had a main effect on root-collar diameter (RCD) up to the 12 weeks (Fig 4B). Root-collar diameter was higher in oak seedlings by 76% than that in maple seedlings for the first week (2.42 ± 0.36 mm and 1.38 ± 0.22 mm, respectively; *F* = 27.07, *P*<0.0001); thereafter, RCD in oak seedlings was higher by 14% for the 12th week (mean ± standard error, 3.39 ± 0.55 mm and 2.97 ± 0.62 mm, respectively; *F* = 5.66, *P* = 0.0004). The spectrum treatment had a main effect on RCD since the second week to the end of the study. Seedlings subjected to the streetlamp spectra treatment had higher RCD than those in the control.

### Biomass accumulation and Nitrogen uptake

**Fig 5:**
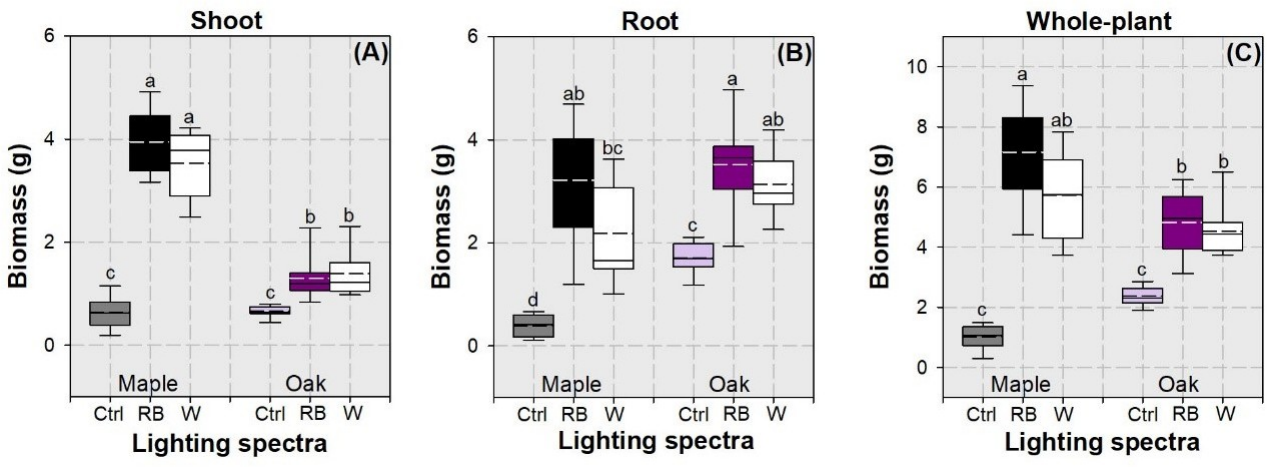
Whisker-box plots for biomass accumulation in shoot (A), root (B), and whole plant (C) in maple (*Acer truncatum* Bunge) and oak (*Quercus mongolica* Fisch. ex Ledeb.) seedlings exposed to simulated streetlamp spectra of red plus blue (RB) and white lights with an un-lighted control. Dash line presents means and full line presents median value. Error bars present 5% and 95% quantiles for upper and lower limits, respectively. Different letters stand for significant different difference across species and lighting spectra using Tukey test at 0.05 level.

Factors of species variation and lighting spectra had an interactive effect on shoot and whole-plant biomasses but did not affect biomass in root (Table 1). Shoot biomass was highest in maple seedlings subjected to the RB and white spectra, followed by that in oak seedlings exposed to the two Streetlamp lighting treatments (Fig 5A). Shoot biomass in controlled seedlings was lowest in both species. The Streetlamp lighting spectrum treatment had a significant effect on root biomass (Table 1). Seedlings in the RB spectrum had highest root biomass (mean ± standard error: 3.37 ± 0.85 g), followed by those in the white spectrum (2.66 ± 0.78 g) (Fig 5B). Controlled seedlings had lowest root biomass (1.05 ± 0.62 g). Whole-plant biomass was highest in maple seedlings subjected to the RB spectrum, followed by that in oak seedlings subjected to the LED treatment, and that in the controlled seedlings was the lowest (Fig 5C).

**Table 1:** *P* values from split-plot designed analysis of variance (ANOVA) of species, light spectra, and their interactive effects on biomass and nitrogen (N) concentration and content in maple (*Acer truncatum* Bunge) and oak (*Quercus mongolica* Fisch. ex Ledeb.) seedlings.

Plant variables	Species	Light	Species × Light
Shoot biomass	**0.0099**	**<0.0001**	**<0.0001**
Root biomass	0.0613	**<0.0001**	0.1237
Whole-plant biomass	0.1931	**<0.0001**	**<0.0001**
Whole-plant N concentration	0.7085	**<0.0001**	**0.0131**
Whole-plant N content	0.3937	**<0.0001**	**<0.0001**

**Fig 6:**
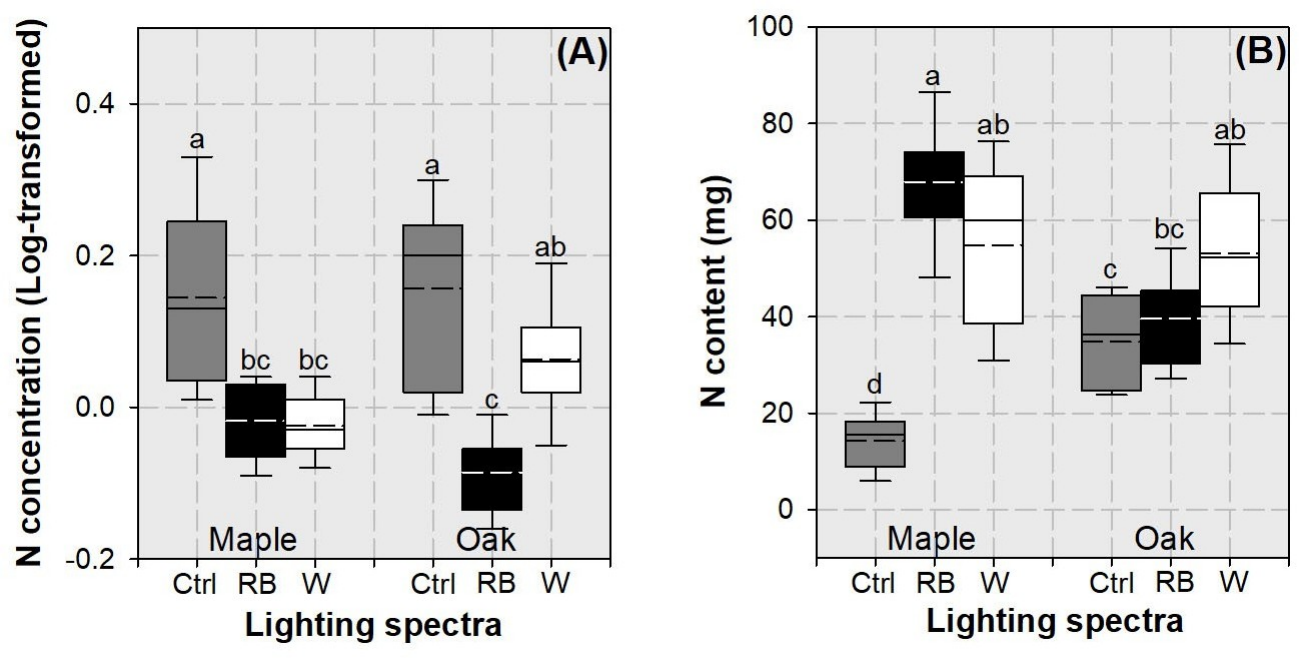
Whisker-box plots for N concentration (A) and N content (B) in whole plant of maple (*Acer truncatum* Bunge) and oak (*Quercus mongolica* Fisch. ex Ledeb.) seedlings exposed to simulated streetlamp spectra of red plus blue (RB) and white lights with an un-lighted control. Dash line presents means and full line presents median value. Error bars present 5% and 95% quantiles for upper and lower limits, respectively. Different letters stand for significant different difference across species and lighting spectra using Tukey test at 0.05 level.

Both N concentration and content at the whole-plant scale were responsive to the interactive effects of species variation and lighting spectra (Table 1). Whole-plant N concentration was highest in controlled seedlings in both species, which was higher than that in maple seedlings subjected to the two LED spectra (Fig 6A). Whole-plant N concentration in oak seedlings subjected to the white light spectrum was higher than that in RB-light treated oak seedlings (Fig 6A). maple seedlings subjected to the RB spectrum had the highest whole-plant N content, followed by seedlings in both species exposed to white light spectrum (Fig 6B). Controlled seedlings had lowest whole-plant N content.

**Fig 7:**
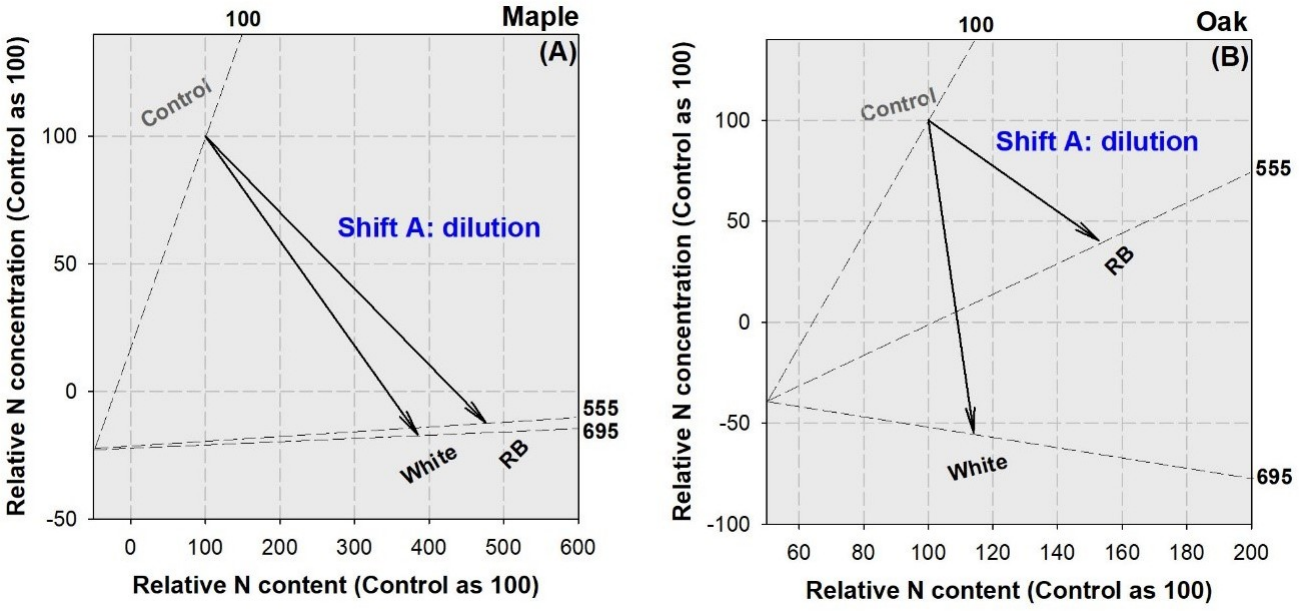
Vector analysis of biomass, N content, and N concentration in whole plant of maple (*Acer truncatum* Bunge) (A) and oak (*Quercus mongolica* Fisch. ex Ledeb.) seedlings (B) exposed to simulated streetlamp spectra of red plus blue (RB) and white lights with an un-lighted control. Biomass has been standardized as values outside the cell frame. Shift A stands for N dilution.

In both maple and oak seedlings, whole-plant biomass increased in both LED spectra treatments relative to that in the control, so did N content (Fig 7). However, whole-plant N concentration declined in LED-treated seedlings for both species, which resulted in a nutritional symptom of dilution.

### Carbohydrate metabolism

**Fig 8:**
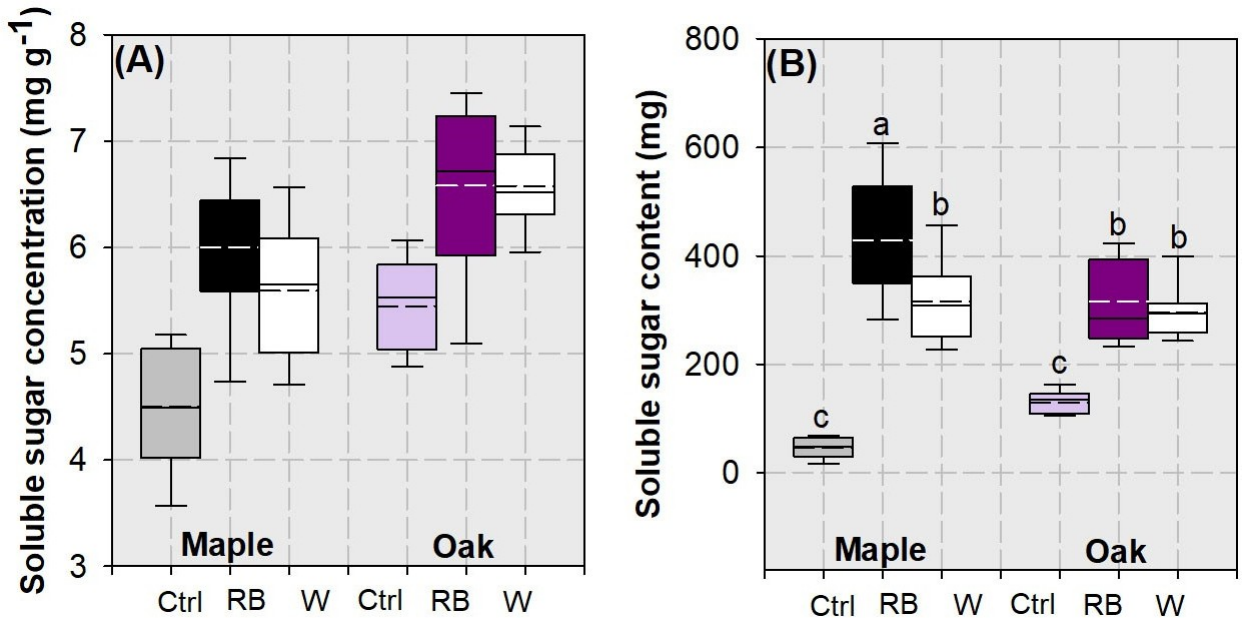
Whisker-box plots for soluble sugar concentration (A) and content (B) in whole plant of maple (*Acer truncatum* Bunge) and oak (*Quercus mongolica* Fisch. ex Ledeb.) seedlings exposed to simulated streetlamp spectra of red plus blue (RB) and white lights with an un-lighted control. Dash line presents means and full line presents median value. Error bars present 5% and 95% quantiles for upper and lower limits, respectively. Different letters stand for significant different difference across species and lighting spectra using Tukey test at 0.05 level.

Factors of species variation and light spectra had a single effect on whole-plant soluble-sugar concentration (Table 2). Compared to sugar concentration in maple (mean ± standard error: 5.37 ± 0.74 mg g^-1^), that in oak seedlings (6.20 ± 0.67 mg g^-1^) was higher by 16% (Fig 8A). Compared to soluble sugar concentration in the control (4.97 ± 0.60 mg g^-1^), that in RB (6.29 ± 0.69 mg g^-1^) and white (6.09 ± 0.62 mg g^-1^) spectra increased by 26% and 22%, respectively (Fig 8A).

**Table 2:** *P* values from split-plot designed analysis of variance (ANOVA) of species, light spectra, and their interactive effects on carbohydrate variables in maple (*Acer truncatum* Bunge) and oak (*Quercus mongolica* Fisch. ex Ledeb.) seedlings.

Carbohydrate variables	Species	Light	Species × Light
Soluble sugar			
concentration	**0.0369** ^1^	**<0.0001**	0.5510
content	0.4440	**<0.0001**	**0.0002**
Starch			
concentration	**0.0013**	**<0.0001**	0.4749
content	**0.0075**	**<0.0001**	0.2005
NSC ^2^			
concentration	**0.0013**	**<0.0001**	0.1293
content	**0.0205**	**<0.0001**	0.2180

Note

^1^ values in bold font highlight significant effect

^2^ NSC, non-structural carbohydrate.

Factors of species variation and lighting spectra had an interactive effect on whole-plant soluble sugar content (Table 2). maple seedlings in the RB spectrum had the highest whole-plant soluble-sugar content, followed by maple seedlings subjected to the white light spectrum and oak seedlings in the Streetlamp lighting spectra (Fig 8B). Controlled seedlings had lowest soluble sugar content at the whole plant scale.

**Fig 9:**
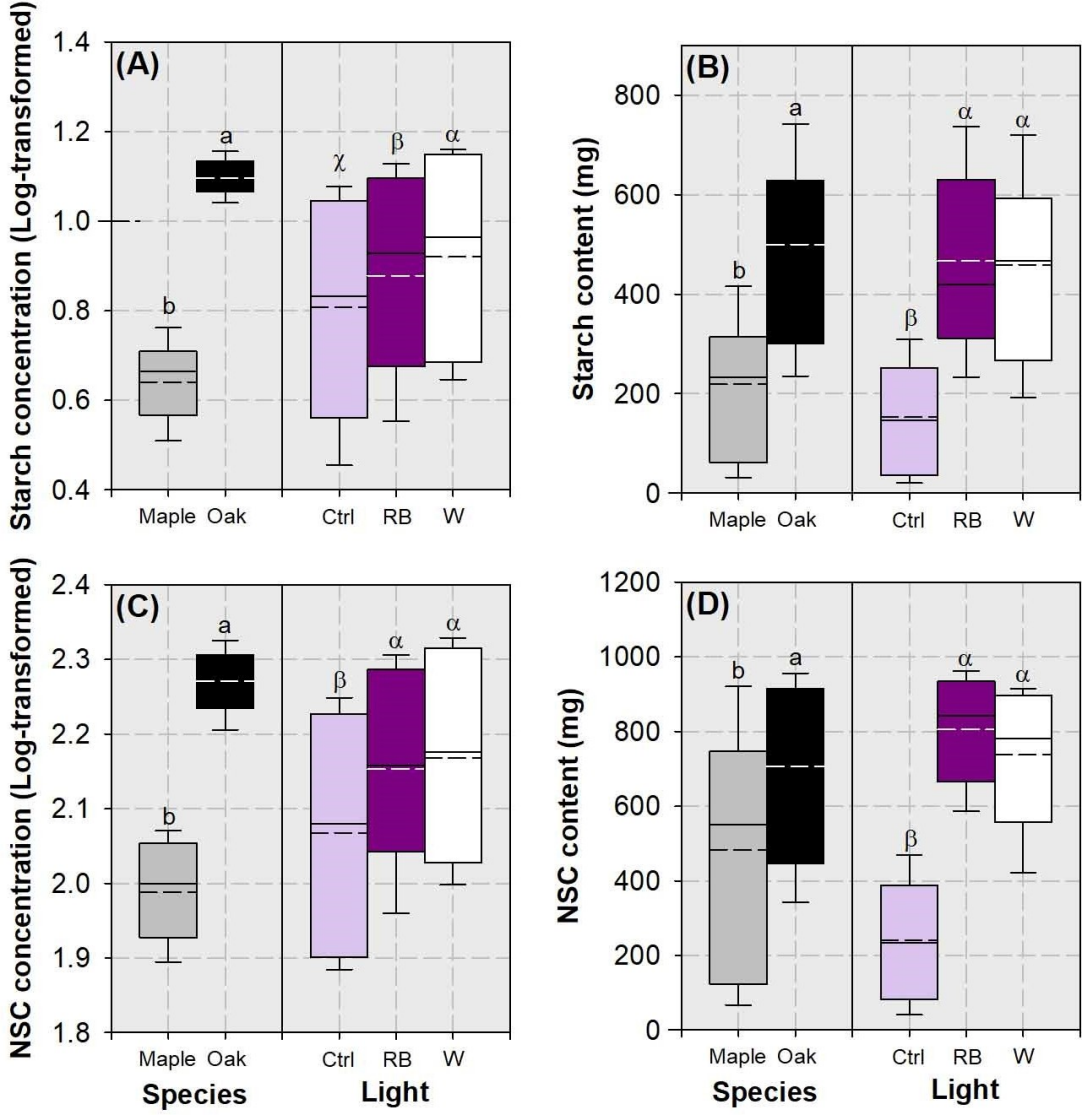
Whisker-box plots for starch concentration (A) and content (B) and nont-structural carbohydrate (NSC) concentration (C) and content (D) in whole plant of maple (*Acer truncatum* Bunge) and oak (*Quercus mongolica* Fisch. ex Ledeb.) seedlings exposed to simulated streetlamp spectra of red plus blue (RB) and white lights with an un-lighted control. Dash line presents means and full line presents median value. Error bars present 5% and 95% quantiles for upper and lower limits, respectively. Different letters stand for significant different difference across species and lighting spectra using Tukey test at 0.05 level. Lower case letters are labelled for species variation; Latinate letters are labelled for spectra variation.

Species variation and light spectra had separated single effect on starch and NSC concentrations and contents (Table 2). Oak seedlings had higher starch and NSC concentrations and contents than maple seedlings (Fig 9). Both LED-spectra treatments can result in higher starch and NSC concentrations and contents compared to the control. In addition, starch concentration was higher in the white light spectrum than in the RB spectrum (Fig 9A).

### Water content ratio

**Fig 10:**
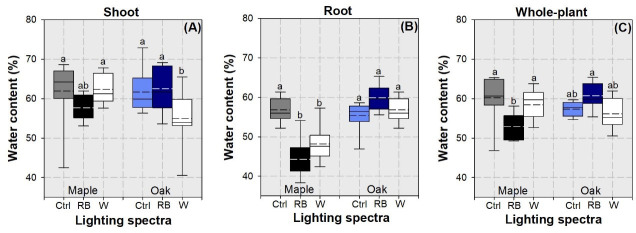
Whisker-box plots for water content ratio in shoot (A), root (B), and whole plant (C) in maple (*Acer truncatum* Bunge) and oak (*Quercus mongolica* Fisch. ex Ledeb.) seedlings exposed to simulated streetlamp spectra of red plus blue (RB) and white lights (W) with an un-lighted control (Ctrl). Dash line presents means and full line presents median value. Error bars present 5% and 95% quantiles for upper and lower limits, respectively. Different letters stand for significant different difference across species and lighting spectra using Tukey test at 0.05 level.

Factors of species variation and lighting spectra had an interactive effect on water content ratio in shoot (*F* = 5.16; *P* = 0.0097), root (*F* = 17.45; *P*<0.0001), and whole-plant (*F* = 11.62; *P*<0.0001). Oak seedlings subjected to the white light spectrum had lower water content in shoot than that in other treated seedlings except that in maple seedlings subjected to the RB spectrum (Fig 10A). maple seedlings subjected to the Streetlamp lighting spectra had lower root water content than other treatments (Fig 10B). Whole-plant water content ratio was lower in RB-light treated maple seedlings than in the controlled maple seedlings and RB-light treated oak seedlings (Fig 10C).

### Nutrient uptake and utilization efficiencies

Maple seedlings subjected to the RB spectrum had the highest *NUE*, followed by oak seedlings treated by white light spectrum (Table 3). The variable of *NUI* for biomass was highest in RB-light treated maple and oak seedlings and white-light treated maple seedlings. Nitrogen utilization index for biomass in oak seedlings subjected to the white light spectrum was lower than that in above-mentioned three treatments but higher than that in controlled seedlings. maple seedlings subjected to the RB-light treatment had highest *NUI* for soluble sugar, while the RB-light treated oak seedlings had the highest *NUI* for starch.

**Table 3:** Split-plot designed analysis of variance (ANOVA) of species (S), light spectra (L), and their interactive effects (S × L) on nitrogen uptake and utilization efficiencies in maple (*Acer truncatum* Bunge) and oak (*Quercus mongolica* Fisch. ex Ledeb.) seedlings.

Species	Light spectra	*NUE* ^1^		*NUI* ^2^							
				Biomass		Soluble sugar		Starch		NSC	
		Mean	SE ^3^	Mean	SE	Mean	SE	Mean	SE	Mean	SE
maple	Ctrl	17.82d ^4^	6.23	0.79c	0.36	35.23d	17.20	31.20d	17.52	66.42	34.61
	RB ^5^	84.86a	12.27	7.65a	2.12	457.69a	137.86	344.72c	85.41	802.40	205.14
	White ^6^	68.58ab	18.54	6.00a	1.21	331.79bc	57.10	307.53c	92.86	639.31	137.39
Oak	Ctrl	43.46c	10.34	1.72c	0.49	93.58d	28.08	193.59c	58.26	287.17	85.81
	RB	49.54bc	9.99	5.94a	1.41	388.47ab	98.02	746.62a	174.79	1135.10	264.57
	White	66.40ab	15.45	3.94b	0.70	257.66c	40.58	546.43b	103.33	804.10	142.76
ANOVA ^7^											
*P* value	S ^8^	0.3939		0.1310		0.3444		0.0128		0.0403	
	L ^9^	< .0001		< .0001		< .0001		< .0001		< .0001	
	S × L	< .0001		0.0030		0.0378		0.0085		0.3794	

Note

^1^ *NUE*, nitrogen uptake efficiency

^2^ *NUI*, nutrient utilization index

^3^ SE, standard error

^4^ different letters in a column indicate significant difference according to Tukey test at 0.05 level

^5^ RB, red and blue lights LED-spectrum

^6^ White, white light LED-spectrum

^7^ ANOVA, analysis of variance

^8^ S, species

^9^ L, lighting spectra.

Factors of species variation and lighting spectra had single effects on *NUI* for NSC (Table 3). Nitrogen utilization index for NSC in oak seedlings (mean ± standard error: 742.12 ± 343.34) was higher than that in maple seedlings (502.71 ± 302.54) by 48% (Table 3). Compared to *NUI* for NSC in controlled seedlings (176.80 ± 115.84), that in seedlings exposed to the RB- (968.75 ± 266.75) and white-lights (721.71 ± 150.40) was higher by 448% and 308%, respectively.

### Priciple component analysis

**Fig 11:**
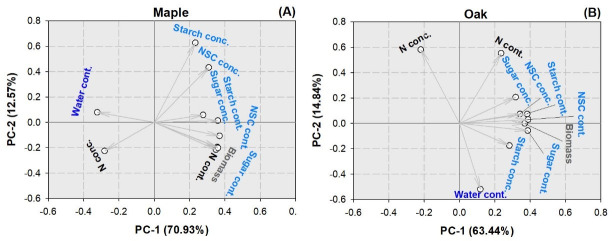
Principle component analysis (PCA) of biomass, nitrogen (N) content (Ncont.) and concentration (Nconc.), soluble sugar content (Sugarcont.) and concentration (Sugar), starch content (Starchcont.) and concentration (Starchconc.), non-structural carbohydrate (NSC) content (NSCcont.) and concentration (NSCconc.), and water content (Watercont.) in maple (*Acer truncatum* Bunge) and oak (*Quercus mongolica* Fisch. ex Ledeb.) seedlings.

For maple seedlings, the first two PCs together account for 83.50% of variation, whereas the first PC accounted for 70.93% and the second 12.57% (Fig 11A). The whole-plant water content had a negative relationship with biomass (*R* = -0.5802, *P* = 0.0015), N content (*R* = -0.5788, *P* = 0.0016), sugar content (*R* = -0.6134, *P* = 0.0007), starch content (*R* = -0.5366, *P* = 0.0039), and NSC content (*R* = -0.5939, *P* = 0.0011). Biomass had a negative relationship with N concentration (*R* = -0.6958, *P*<0.0001), but its relationship with N content was positive (*R* = 0.9776, *P* = 0.0001). Biomass also had a positive relationship with sugar concentration (*R* = 0.6065, *P* = 0.0008), sugar content (*R* = 0.9758, *P*<0.0001), starch concentration (*R* = 0.4206, *P* = 0.0289), starch content (*R* = 0.9360, *P*<0.0001), NSC concentration (*R* = 0.6276, *P* = 0.0005), and NSC content (*R* = 0.9828, *P*<0.0001). N concentration had a negative relationship with sugar concentration (*R* = -0.4383, *P* = 0.0222), sugar content (*R* = -0.6769, *P* = 0.0001), starch concentration (*R* = -0.5884, *P* = 0.0012), starch content (*R* = -0.6789, *P*<0.0001), NSC concentration (*R* = -0.6197, *P* = 0.0006), and NSC content (*R* = -0.6955, *P*<0.0001). In contrast, N content had a positive relationship with sugar concentration (*R* = 0.6012, *P* = 0.0009), sugar content (*R* = 0.9538, *P*<0.0001), starch concentration (*R* = 0.4163, *P* = 0.0308), starch content (*R* = 0.9229, *P*<0.0001), NSC concentration (*R* = 0.6174, *P* = 0.0006), and NSC content (*R* = 0.9644, *P*<0.0001).

For oak seedlings, the first two PCs together account for 78.28% of variation, whereas the first PC accounted for 63.44% and the second 14.84% (Fig 11B). Biomass had a negative relationship with N concentration (*R* = -0.5551, *P* = 0.0027), but its relationship with N content was positive (*R* = 0.6336, *P* = 0.0004). In addition, biomass had a positive relationship with sugar concentration (*R* = 0.4647, *P* = 0.0146), sugar content (*R* = 0.9590, *P*<0.0001), starch concentration (*R* = 0.6018, *P* = 0.0009), starch content (*R* = 0.9806, *P*<0.0001), NSC concentration (*R* = 0.6279, *P* = 0.0005), and NSC content (*R* = 0.9834, *P*<0.0001). N concentration had a negative relationship with sugar concentration (*R* = -0.4148, *P* = 0.0314), sugar content (*R* = -0.5693, *P* = 0.0019), starch content (*R* = -0.5163, *P* = 0.0058), NSC concentration (*R* = -0.4020, *P* = 0.0376), and NSC content (*R* = -0.5395, *P* = 0.0037). In contrast, N content had a positive relationship with sugar content (*R* = 0.5834, *P* = 0.0014), starch concentration (*R* = 0.4574, *P* = 0.0165), starch content (*R* = 0.6665, *P* = 0.0001), NSC concentration (*R* = 0.4263, *P* = 0.0266), and NSC content (*R* = 0.6453, *P* = 0.0003).

## Discussion

### Effect of LED spectra on growth and biomass in maple and oak seedlings

The PPFD of 80 μmol m^-2^ s^-1^ is lower than that in ordinary sunlight as needed for the growth of temperate trees. However, our PPFD was higher than that in several other studies of artificial lighting on tree seedlings which was around 60–70 μmol m^-2^ s^-1^ [6–8]. Because the typical canopy height of a street tree is about 6–9 m [5, 29], the most part of canopy may have been exposed to low light condition. The PPFD from streetlamp lighting for street trees in Florence was 7–12 μmol m^-2^ s^-1^ [5], and that for trees in Finland was 1.3 μmol m^-2^ s^-1^ [29]. The light intensity from streetlamp lights was only about 1% of that from local sunlight [29]. The low light intensity that was tested on lower parts of tree canopy has been found to affect phenology. Therefore, the lighting intensity that was tested in this study was high enough for a study to elicit physiological response of tree seedlings.

We found that the simulated streetlamp spectra can promote the above-ground elongation in maple seedlings but the spectra impact on oak seedlings was not effective. Our results about maple seedlings appear to be rarely supported by other findings in current literature. Singh et al. [42] reported spectral effect on micropropagation of sugar maple (*Acer saccharum* Marsh.) at low light intensity. For oak seedlings in our study, however, neither height nor RCD showed any significant response to the spectra treatment. This part of findings disagrees to those found by Astolfi et al. [43], where height growth of oak seedlings varied between contrasting spectra caused by fluorescent and LED lights. Above-results together resulted in a species-specific response of height to the spectra treatment with no difference of RCD between species. The species-specific response of growth to artificial lighting was also reported across *Picea abies*, *Pinus sylverstris*, and *Quercus ilex* seedlings [44]. The species-specific effect of spectra on tree growth may widely exist for different species, which needs more evidence to confirm the specific response in further work.

Our biomass results followed the general trend of height growth, which was increased by the extended LED lighting, no matter what spectra were employed, compared to the untreated control. Results from previous studies concur with ours by no change of biomass in forest plants between LED and HPS spectra [6, 7]. However, tree seedling biomass was found to vary among different LED spectra in different proportions of red, green, and blue lights [6, 8]. These studies, plus ours, together showed a high variation of biomass that are randomly caused by lighting spectra. The lack of difference for NSC between LED spectra concurs with that for biomass and the positive relationship between NSC and biomass. Statistics indicated that biomass in shoot and whole plant showed no difference between spectra. Root biomass also showed the same results when data were compared by a species × spectra combination. Root biomass was also found to be null to the spectra variation in *Dalbergia odorifera* seedlings although shoot biomass responded [8]. Therefore, the lack of effect on biomass allocation can also be the reason leading to root biomass. The increase in biomass following LED lighting can also result from the promotion on dry mass accumulation in a prolonged photoperiod which has been reported on several other species [15, 45]. However, our results about biomass oppose those in studies where biomass was one of the most changeable variables among different lighting qualities [6–9]. Overall, trees exposed to streetlamp lighting can obtain accelerated growing appearance, but the growth and biomass responses would not vary to different light spectra.

### Nutritional response in maple and oak seedlings to LED spectra

The lower N concentration in LED lighting compared to the control was interpreted as a symptom of dilution, which was simultaneously caused by the decline in whole-plant N content and biomass. Streetlamp lighting can accelerate N utilization for growth and result in the pool of diluted N. Nutrient dilution usually accompanied with accelerated growth in a prolonged photoperiod [15, 16] or in some spectral wavelength [6, 7]. Although seedlings were fed with a rate of N input to simulate deposition, the heavy loading did not alleviate N dilution.

We found lower N concentration in RB spectrum than in white light spectrum for oak seedlings but not for maple seedlings. This was because RB spectrum caused higher consumption of N for utilization in oak than in maple. Although RB spectrum failed to promote growth or biomass in oak seedlings compared to the white one, the RB spectrum increased utilization of N for biomass and starch productions. Correlation indicated that N concentration had a negative relationship with biomass while biomass had a positive relationship with starch for both species; hence the depletion of N as decline in concentration was the result of utilization to produce biomass through starch accumulation. As we did not take N deposition as a source of effects, it was unclear whether simulated N deposition imposed any impact on utilization.

The RB spectrum can increase N content compared to the control in maple seedlings, but the case was not found in oak seedlings. The white light spectrum, however, can promote N content in seedlings from both species. At least three explanations can be responsible for the species-specific response of N uptake to different spectra. Firstly, the photosynthetic activity is associated with the demand for N assimilation by leaves which is accompanied by a large amount of photosynthetic-involved processes [46]. Given the fact that maple and oak have different net photosynthesis rates [47], the demand for N uptake and allocation is easily to be diverged between the two species. Secondly, chlorophyll content was found to be highly varied in leaves of different species [48] and the variation of pigment may have driven the difference of need to absorb N. Finally, activities of nitrate reductase and nitrite reductase have been found to be changed by light quality [49, 50], the genetic difference of N assimilation by coding the nitrate reductase genes in the two species determined the difference of foliar N content [51]. Our results concur with those about *NUE* in response to treatments. Thus, the white light spectrum can promote N uptake for both species, but the RB spectrum can only promote N uptake in maple seedlings. Given that nutrient content is the product of nutrient concentration and biomass, a spectrum that can benefit both biomass and content increases can also promote the uptake [9]. As N content was positively correlated with biomass, the increase of N content went with the biomass accumulation despite the decline of N concentration. Correlation analysis indicated that, at the same level of biomass, oak seedlings would uptake more N and stored as greater content than maple seedlings, which suggests a better ability of oak to seize N compared to maple. Overall, N dilution is an inevitable weakness for trees under streetlamps. The white light spectrum is better than that from RB-wavelengths because of the promotion on N uptake especially for oak.

### Response of carbohydrates in maple and oak seedlings to LED spectra

In our study, oak seedlings had higher sugar and starch concentrations, subsequently an overall higher concentration of NSC, relative to maple seedlings. The outperformance of NSC in oak compared to that in maple was also found by Kruger and Reich [52]. Lei and Koike [53] found a higher photosynthetic rate in full light but a slower consumption of carbohydrate in shade for oak and maple, which together resulted in higher accumulation of NSC. Our seedlings were subjected to LED lighting in an extended photoperiod. This stimulated photosynthesis and carbohydrate production in oak seedlings with a higher efficiency and shaped the results that more carbohydrate reserved in oak seedlings.

The highest sugar content in maple seedlings subjected to the RB light spectrum among all treatments was quite similar to the biomass accumulation in above-ground and whole-plant parts. Soluble sugar content had a close relationship with biomass for both species. Therefore, it was the biomass accumulation that drove the increase of soluble sugar content in RB-light treated maple seedlings. Starch concentration was higher in white light spectrum than in RB light and the control. In contrast, starch concentration was not affected by LED lighting between different spectra for *Dalbergia odorifera* [8]. Our starch concentration showed the only difference for all carbohydrates in response to the two different spectra. This was shaped by combined involvements of biomass and N concentration, which had a positive and negative relationship with starch concentration, respectively. Therefore, our results concur to those found by Li et al. [8] that the two LED spectra cannot result in a general variation of carbohydrates. This went in accordance with the result for biomass and together promote to a conclusion that streetlamp spectra can unlikely cause the response of dry mass and NSC productions in maple and oak tree seedlings.

The positive relationship between carbohydrate concentration and biomass is because of the synchronization of dry mass and carbohydrates from the photosynthetic production. The negative relationship between carbohydrate and N concentration is a common phenomenon that exists in trees [54, 55]. To assimilate N needs the consumption of starch and hydrolyzation of soluble sugars to supply the frame of carbon skeleton and energy. According to the relationships between carbohydrates with biomass and N concentration, with the increase of carbohydrates oak is the species with a lower rate of biomass accumulation but a higher rate of N concentration decline compared to maple. These results suggest that, exposed to the streetlamp lighting, oak uses carbohydrates slowly but at a high N cost while maple chooses to use the contrasting strategy. The exposure to streetlamp lighting can generally promote the carbohydrate production and accumulation in oak and maple trees but the spectra effect was rare.

### Response of water content in maple and oak seedlings to LED spectra

Our study revealed that both biomass and carbohydrates had negative relationships with water content in maple seedlings but not in oak seedlings. These meant that the process of producing and accumulating biomass and carbohydrate was accompanied by the consumption of tissue water. Lighting spectra can promote the gas exchange to elevate C assimilation for carbohydrate and biomass accumulation, which would decrease WUE through increasing transpiration [56, 57]. The spectrum from RB light in our study resulted in a decline of whole-plant water content in maple seedlings compared to the control. These results concur with those found on vegetative and flower crops [56, 57]. In addition, a white light spectrum was found to indicate an increase in transpiration rate and internal CO_2_ concentration and a subsequent decrease in WUE in tomato (*Solanum lycopersicum*) plants. The white light spectrum was also found to decrease water content in the shoot part of oak seedlings. This was contradicted to the results that no relationship existed between whole-plant carbohydrate concentration or biomass and water content in oak seedlings. However, the difference of water content in roots of oak seedlings between the control and the white light treatments was neither apparent nor significant. We surmise that, when exposed to streetlamps, oak seedlings did not have as enlarged change in stomatal conductance as maple seedlings which resulted in the null response of water content in oak. Future work is suggested to further detect WUE and stomatal exchange in street tree leaves to reveal the mechanism of water content in response to streetlamp lights.

## Conclusions

Most significant results in this study were aroused between artificial lighting, no matter what spectra it was, and the un-lighted control. The effect of streetlamp lighting imposed on street trees were mainly derived from an extended photoperiod instead of spectrum. Maple is a light-responsive species that can obtain promotion on growth at a low cost of N and water. In contrast, oak is a reserved species that reserved the use of carbohydrate in response to streetlamp lighting. Therefore, the color of streetlamps unlikely cause varied effects on street trees. According to the different phenotypes, maple can be used as the chosen species for newly planted stocks to green the streetlamp landscape with big canopy in a shorter time. Oak can be used along with streetlamps as a reserved and stable species that do not need frequent budget to trim the growing crown that can be enlarged by night lighting. This study is just an initiator that can encourage new work to detect more spectra from streetlamps from more regions in the world. More tree species can be tested from different urban ecosystem biomes like evergreen broadleaf in sub-tropical regions and coniferous in boreal parts.

## Supporting information

S1 Raw data (XLSX)
